# Evaluation of Translation Rate Through L-azidohomoalanine (AHA) Incorporation and Subsequent Alkyne Fluorophore–Mediated Click Chemistry in Yeast

**DOI:** 10.21769/BioProtoc.5379

**Published:** 2025-07-20

**Authors:** Mainak Pratim Jha, Koyeli Mapa

**Affiliations:** Protein Homeostasis Laboratory, Department of Life Sciences, School of Natural Sciences, Shiv Nadar Institution of Eminence, Delhi-NCR, India

**Keywords:** Click chemistry, L-azidohomoalanine (AHA), Protein translation, *Saccharomyces cerevisiae*, Flow cytometry, Confocal microscopy, SDS-PAGE, Fluorescence gel imaging.

## Abstract

Accurate measurement of protein translation rates is crucial for understanding cellular processes and disease mechanisms. However, existing methods for quantifying translation rates in yeast cells are limited. Here, we present a streamlined protocol for measuring protein translation rates in *Saccharomyces cerevisiae* using the methionine analog L-azidohomoalanine (AHA), which is the L isoform of this synthetic amino acid, and fluorophore-labeled alkyne dye-based Click chemistry. Our method involves incorporating AHA into newly synthesized proteins, followed by detection using confocal microscopy, flow cytometry, and SDS-PAGE. We validated our protocol by measuring translation rates under various stress conditions, including heat stress, endoplasmic reticulum (ER) stress induced by tunicamycin, and translation inhibition by cycloheximide. Confocal microscopy revealed differential AHA incorporation and fluorescence intensity across conditions. Flow cytometry quantitatively confirmed significant increases in translation rates under heat stress and decreases under ER stress compared to unstressed conditions at 6 and 24 h post-treatment. Imaging of gels under fluorescence detectors following SDS-PAGE further visualized newly synthesized proteins, with no detectable translation after cycloheximide treatment. Our protocol offers enhanced precision and selectivity compared to existing methods for mammalian cells and represents the first standardized approach for measuring translation rates in yeast. Despite limitations in required specialized equipment and expertise, this method holds promise for diverse applications in biotechnology and biomedical research, enabling investigations into protein synthesis regulation in yeast systems.

Key features

• This study presents the first standardized protocol for measuring protein translation in budding yeast using AHA and Click chemistry, addressing yeast-specific challenges effectively.

• The study uses microscopy, flow cytometry, and fluorescence gel imaging to robustly validate yeast translation rates, ensuring reliable, reproducible results across cellular and biochemical levels.

• The method detects translation changes under stress: increased with heat, decreased with ER stress, and halted by cycloheximide, highlighting its sensitivity for proteostasis research.

• Despite requiring specialized equipment and expertise, the method offers valuable applications in biomedical research, metabolic engineering, and drug screening focused on protein homeostasis in yeast.

## Graphical overview


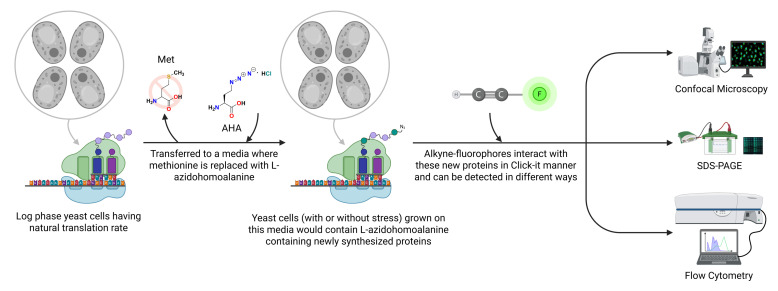


**Schematic overview of the L-azidohomoalanine (AHA)-based Click chemistry protocol to measure translation rate in *Saccharomyces cerevisiae*.** Schematic representation showing L-azidohomoalanine incorporated into newly synthesized proteins that can be detected by using alkyne-modified fluorophores and qualitatively and quantitatively measured by various techniques, as depicted in the figure.

## Background

Protein translation is a critical cellular process that converts genetic information from DNA into functional proteins, which are essential for various cellular activities such as biochemical catalysis, structural integrity, and intercellular signaling. This process involves the conversion of messenger RNA (mRNA) into proteins through tightly regulated stages, including initiation, elongation, termination, and ribosome recycling, thereby ensuring accurate protein synthesis in response to internal and external stimuli [1]. Precision in protein production is vital for cellular function and organismal homeostasis, with aberrations potentially leading to dysfunctional proteins and pathological conditions [2].

Regulation of protein translation, which is crucial for cellular equilibrium and environmental adaptation, involves mRNA structure, translation factors, and aminoacyl-tRNA. The 5'-UTR of mRNA contains motifs that regulate translation stages, while secondary structures and sequences affect ribosomal access. Initiation factors assemble the translation complex, elongation factors add amino acids, and release factors terminate the synthesis. Aminoacyl-tRNAs deliver amino acids, affecting translation rate and efficiency [2]. Under stress conditions, cells store mRNA in stress granules and P-bodies [3]. Disrupted protein translation regulation is central to diseases such as cancer, neurodegenerative disorders, and metabolic disorders owing to altered translation factors, tRNA availability, or ribosome defects. Neurodegenerative diseases involve the accumulation of misfolded proteins. Advances in ribosome profiling and mass spectrometry have enhanced the study of translation errors and their impact on disease, suggesting new therapeutic approaches [4].

Ribosomes can simultaneously translate multiple mRNAs to form polysomes and enhance their efficiency. The movement of mRNA through the ribosome affects the speed of protein synthesis, with suboptimal mRNA structures hindering ribosome binding and movement, thus reducing efficiency [3]. Translation rates depend on the translational machinery and factors such as nutrient availability and stress responses. Nutrient abundance supplies essential components for protein synthesis, whereas nutrient scarcity can halt translation. Stress can sequester mRNA in stress granules, reducing the available mRNAs for translation and conserving energy. During stress, translation regulation enables cells to balance protein synthesis with immediate needs, prioritize critical functions, and optimize survival in changing environments [2,5].

**Figure 1.**
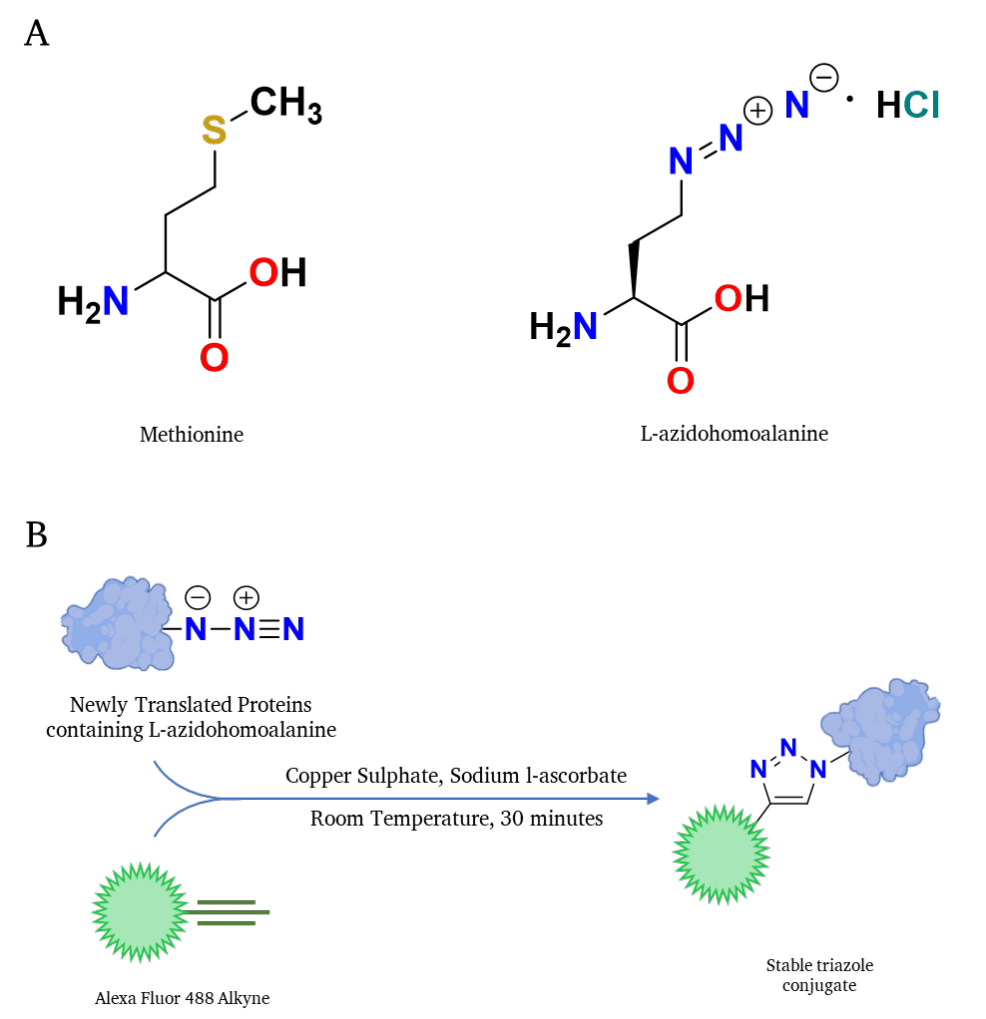
Schematic representation of the translation rate determination through Click chemistry–based reaction. (A) Structure of methionine is shown on the left, and the structure of the methionine analog L-azidohomoalanine is shown on the right. (B) L-azidohomoalanine, incorporating newly synthesized proteins, interacts with the alkyne-containing fluorophore in a Click-IT reaction fashion; subsequently, the fluorophore-labeled newly synthesized proteins can be detected in any fluorescence-detection system.

This study presents a streamlined methodology for accurately measuring the protein translation rate in the budding yeast *Saccharomyces cerevisiae* using a methionine analog, L-azidohomoalanine (AHA), and subsequent fluorophore-labeled alkyne dye-based Click chemistry reaction (Figure 1). While Click chemistry–based detection techniques are available for mammalian cells, none address the complexities associated with budding yeast samples. Our protocol delineates the detailed step-by-step process of determining the protein translation rate in budding yeast; our results were validated by incorporating L-azidohomoalanine into newly synthesized proteins using flow cytometry, microscopy, and visualization in protein gels. In essence, our protocol effectively establishes a convenient method for evaluating protein translation rate in budding yeast under various growth conditions.

## Materials and reagents

**Biological materials**

1. Yeast strain BY4741 (genotype: MATa his3Δ1 leu2Δ0 met15Δ0 ura3Δ0; parent strain: S288C; source: S288C/EUROSCARF)

**Reagents**

1. Glucose (Sigma, catalog number: G8270)

2. Yeast nitrogen base (Himedia, catalog number: M151)

3. Ammonium chloride (MP Biomedicals, catalog number: 150109)

4. Methionine (Sigma, catalog number: M9625)

5. Phenylalanine (Sigma, catalog number: P5482)

6. Adenine (Sigma, catalog number: A2786)

7. Arginine (Sigma, catalog number: A8094)

8. Threonine (Sigma, catalog number: T8441)

9. Lysine (Sigma, catalog number: L9037)

10. L-azidohomoalanine (Sigma, catalog number: 900892)

11. Histidine (Sigma, catalog number: H5659)

12. Leucine (Sigma, catalog number: L8912)

13. Tryptophan (Sigma, catalog number: T8941)

14. Uracil (Sigma, catalog number: U1128)

15. Agar powder, bacteriological grade (Himedia, catalog number: GRM026)

16. Tunicamycin (Sigma, catalog number: T7765)

*Note: Light sensitive, store at -20 °C, prepare dilutions right before use as a stressor in cultures.*

17. Cycloheximide (Sigma, catalog number: C4859)

*Note: Light sensitive, store at -20 °C, prepare dilutions right before use as a stressor in cultures.*

18. Sodium hydroxide (MP Biomedicals, catalog number: 153495)

19. Sodium chloride (MP Biomedicals, catalog number: 102892)

20. Potassium chloride (MP Biomedicals, catalog number: 151944)

21. Sodium phosphate dibasic (MP Biomedicals, catalog number: 191440)

22. Potassium phosphate dibasic (MP Biomedicals, catalog number: 191431)

23. Hydrochloric acid (Sigma, catalog number: H9892)

24. Tris (MP Biomedicals, catalog number: 194855)

25. EDTA disodium dihydrate (MP Biomedicals, catalog number: 194822)

26. Phenylmethylsulfonyl fluoride (PMSF) (MP Biomedicals, catalog number: 195381)

27. Sodium dodecyl sulfate (MP Biomedicals, catalog number: 811033)

28. Glycerol (MP Biomedicals, catalog number: 193996)

29. Bromophenol blue (MP Biomedicals, catalog number: 805732)

30. β-Mercaptoethanol (MP Biomedicals, catalog number: 190242)

31. Acid-washed glass beads (Sigma, catalog number: G8772)

32. Molecular biology grade absolute ethanol (Hayman premium grade, 100%, catalog number: F205855)

33. Alexa-Fluor 488 alkyne (ThermoFisher, catalog number: A10267)

*Note: Light sensitive, store at -20 °C, prepare dilutions right before use as a Click reagent for permeabilized cells.*

34. Copper (II) sulphate pentahydrate (Sigma, catalog number: C8027)

35. Sodium L-ascorbate (Sigma, catalog number: 11140)

36. Liqui-gel, premixed acrylamide/bis-acrylamide solution (MP Biomedicals, catalog number: 800803)

**Solutions**

1. Synthetic complete media (see Recipes)

2. Synthetic complete media with L-azidohomoalanine (see Recipes)

3. PBS 10× stock (see Recipes)

4. Yeast permeabilization buffer (see Recipes)

5. Click reaction buffer (see Recipes)

6. Sodium hydroxide solution 0.3 M (see Recipes)

7. Protein storage buffer (see Recipes)

8. SDS loading dye 5× stock (see Recipes)

**Recipes**

**1. Synthetic complete media**

**Table:** 

Reagent	Final concentration	Quantity or volume
Glucose	20 mg/mL	10 mL of 200 mg/mL stock
Yeast nitrogen base	1.7 mg/mL	10 mL of 17 mg/mL stock
Ammonium chloride	5 mg/mL	10 mL of 50 mg/mL stock
Methionine	0.02 mg/mL	1 mL of 2 mg/mL stock
Phenylalanine	0.05 mg/mL	1 mL of 5 mg/mL stock
Adenine	0.03 mg/mL	1 mL of 3 mg/mL stock
Arginine	0.03 mg/mL	1 mL of 3 mg/mL stock
Threonine	0.15 mg/mL	1 mL of 15 mg/mL stock
Lysine	0.05 mg/mL	1 mL of 5 mg/mL stock
Histidine	0.02 mg/mL	1 mL of 2 mg/mL stock
Leucine	0.1 mg/mL	1 mL of 10 mg/mL stock
Tryptophan	0.05 mg/mL	1 mL of 5 mg/mL stock
Uracil	0.02 mg/mL	1 mL of 2 mg/mL stock
Autoclaved sterile water	n/a	Up to 100 mL
Total	n/a	100 mL

**2. Synthetic complete media with L-azidohomoalanine**

**Table:** 

Reagent	Final concentration	Quantity or volume
Glucose	20 mg/mL	10 mL of 200 mg/mL stock
Yeast nitrogen base	1.7 mg/mL	10 mL of 17 mg/mL stock
Ammonium chloride	5 mg/mL	10 mL of 50 mg/mL stock
L-azidohomoalanine	0.02 mg/mL	1 mL of 2 mg/mL stock
Phenylalanine	0.05 mg/mL	1 mL of 5 mg/mL stock
Adenine	0.03 mg/mL	1 mL of 3 mg/mL stock
Arginine	0.03 mg/mL	1 mL of 3 mg/mL stock
Threonine	0.15 mg/mL	1 mL of 15 mg/mL stock
Lysine	0.05 mg/mL	1 mL of 5 mg/mL stock
Histidine	0.02 mg/mL	1 mL of 2 mg/mL stock
Leucine	0.1 mg/mL	1 mL of 10 mg/mL stock
Tryptophan	0.05 mg/mL	1 mL of 5 mg/mL stock
Uracil	0.02 mg/mL	1 mL of 2 mg/mL stock
Autoclaved sterile water	n/a	Up to 100 mL
Total	n/a	100 mL

**3. PBS 10× stock**

**Table:** 

Reagent	Final concentration	Quantity or volume
Sodium chloride	80 mg/mL	8,000 mg
Potassium chloride	2 mg/mL	200 mg
Sodium phosphate dibasic	17.8 mg/mL	1,780 mg
Potassium phosphate dibasic	2.4 mg/mL	240 mg
Hydrochloric acid	to adjust pH to 7.4	As required from 100% stock
Autoclaved sterile water	n/a	Up to 100 mL
Total	n/a	100 mL

*Note: Dilute to 1× with sterile distilled water as needed.*

**4. Yeast permeabilization buffer**

**Table:** 

Reagent	Final concentration	Quantity or volume
Absolute ethanol	53%	53 mL of 100% stock
PBS	1×	10 mL of 10× stock
Autoclaved sterile water	n/a	Up to 100 mL
Total	n/a	100 mL

**5. Click reaction buffer**

**Table:** 

Reagent	Final concentration	Quantity or volume
Tris-Cl	2,000 mM	5 mL of 4 M pH 8.5 stock
Copper sulphate	50 mM	0.5 mL of 1 M stock
Alexa-Fluor 488 alkyne	1 μg/mL	10 μL of 1 mg/mL stock
Ascorbic acid	500 mM	1.667 mL of 3 M stock
Autoclaved sterile water	n/a	Up to 10 mL
Total	n/a	10 mL

**6. Sodium hydroxide solution 0.3 M**

**Table:** 

Reagent	Final concentration	Quantity or volume
Sodium hydroxide	300 mM	1,200 mg
Autoclaved sterile water	n/a	Up to 100 mL
Total	n/a	100 mL

**7. Protein storage buffer**

**Table:** 

Reagent	Final concentration	Quantity or volume
Tris-Cl	10 mM	1 mL of 1 M pH 7.4 stock
EDTA	1 mM	0.2 mL of 0.5 M pH 8 stock
Potassium chloride	150 mM	3.75 mL of 4 M stock
PMSF	1 mM	0.5 mL of 200 mM stock
Autoclaved sterile water	n/a	Up to 100 mL
Total	n/a	100 mL

**8. SDS loading dye 5× stock**

**Table:** 

Reagent	Final concentration	Quantity or volume
Tris-Cl	250 mM	25 mL of 1 M pH 6.8 stock
Sodium dodecyl sulfate	4%	20 mL of 20% stock
Glycerol	30%	30 mL of 100% stock
Bromophenol blue	0.6 mg/mL	60 mg
β-Mercaptoethanol	16%	16 mL of 100% stock
Autoclaved sterile water	n/a	Up to 100 mL
Total	n/a	100 mL

**Laboratory supplies**

1. Micropipette tips: 10 μL, 100 μL, and 1,000 μL (Tarsons, catalog numbers: 521010, 521010, and 521020, respectively)

2. Petri dishes (Tarsons, catalog number: 460030)

3. 1.5 mL microcentrifuge tubes (Tarsons, catalog number: 500010)

4. 50 mL centrifuge tubes (Tarsons, catalog number: 546041)

5. Screw-capped glass flasks of 25 and 100 mL (Borosil, catalog numbers: 1501009 and 5021016, respectively)

6. Single-channel pipets, 0.2–2 μL (P2), 2–20 μL (P20), 20–200 μL (P200), 100–1,000 μL (P1000) (Gilson, PIPETMANS, catalog numbers: F144054M, F144056M, F144058M, and F144059M, respectively)

## Equipment

1. Autoclave (ALP, catalog number: 32SDP)

2. Shaker incubator (Scigenics Biotech Orbitek, catalog number: LE-BT-AH)

3. Spectrophotometer (Biospectrometer, Eppendorf, catalog number: 6136000.002)

4. Centrifuges for 50 mL tubes and 1.5 mL microcentrifuge tubes (Eppendorf 5810R and 5425R, catalog numbers: EP022627066 and EP5406000046, respectively)

5. Bead-Ruptor (Omni International, catalog number: 25-010)

6. Laser scanning confocal microscope (Nikon, model: A1R MP+ Multiphoton Advanced Resolution)

7. Fluorescence activated cytometer (Beckman Coulter, model: Cytoflex S)

8. SDS-PAGE unit (Bio-Rad, model: Mini-PROTEAN Tetra Vertical Electrophoresis Cell)

9. Fluorescence-enabled Gel Doc system (Invitrogen, model: iBright)

10. Gel Doc system (Bio-Rad, model: Gel Doc EZ imager, catalog number: 170-8270)

## Software and datasets

1. Nikon NIS-Elements AR (Nikon, 5.20.02); available with the microscope; otherwise, requires a license

2. CytExpert (Beckman Coulter, 2.5.0.77); available with the fluorescence analyzer; otherwise, requires a license

3. FlowJo (FlowJo LLC, 10.8); requires a license

4. GraphPad Prism (GraphPad Software, 9.5.0); requires a license

5. Biorender portal (Biorender, https://app.biorender.com/); requires a license

6. MS Office suite, Excel, PowerPoint, and Word (Microsoft, Office 365); requires a license

## Procedure

**A. Culturing yeast cells and treatment with stressors**

This protocol determines the translation rate of yeast cells under physiological and stressful conditions. We used the BY4741 strain, which usually serves as the wild-type yeast strain throughout all the experiments described here. The detailed protocol is as follows:

1. Take out the glycerol stab of the wild-type strain from the -80 °C fridge and streak on a synthetic complete agar plate (see note below). Incubate the plate for 4–5 days at 30 °C to generate distinct single colonies. After that, keep the plate at 4 °C for future use.

**Pause point:** At this juncture, the strains may be securely stored until the commencement of the experiment. However, it is recommended to re-streak the colonies onto a fresh synthetic-complete agar plate if the original streak plate is older than one month.

*Note: The preparation of synthetic complete agar plates closely resembles the procedure for synthetic complete media (liquid), with the primary distinction being the requirement to dissolve agar powder in water. This mixture must be autoclaved to ensure sterility. Subsequently, the other components, which have been previously sterilized, are combined, and the final volume is adjusted with sterile water as specified in the recipe section. It is essential to thoroughly mix all components and pour the solution into Petri dishes while it remains warm and in liquid form. Once solidified, the agar plates are ready for use in culturing.*

2. Inoculate a single colony of the wild-type strain in 5 mL of synthetic complete media in a 25 mL capped glass flask and grow overnight at 30 °C in a shaker incubator at 200 rpm. This will serve as the primary culture.

**Pause point:** At this stage, it is imperative that all necessary media and reagents are meticulously prepared in advance prior to proceeding.

3. The next morning, from the overnight-grown primary culture, inoculate a total of four secondary cultures in 25 mL of synthetic complete media in 100 mL capped conical flasks at OD_600_ of 0.1. Incubate in the shaker incubator at 200 rpm for approximately 5 h until it reaches 0.4–0.6 OD_600_. Designate each of the four flasks to be used for the *Untreated control, Heat stress condition, Tunicamycin-induced chronic ER stress condition*, and *Cycloheximide-induced translational block stress condition*.

4. At this actively growing log phase, transfer the yeast cells to 50 mL centrifuge tubes and spin down at 3,500× *g* for 5 min at room temperature.

5. Discard the resultant supernatant, resuspend the cells in sterile autoclaved water, and centrifuge again at 3,500× *g* for 5 min at room temperature.

6. Again, discard the resultant supernatants, but this time resuspend the cells in synthetic complete media where methionine is replaced with L-azidohomoalanine in the same proportion as with normal media. Transfer to fresh 100 mL capped conical flasks.

**Critical point:** At this juncture, it is imperative that the new medium in which the pelleted cells are to be resuspended is devoid of any methionine and instead contains L-azidohomoalanine as a substitute. The accurate measurement of the rate of translation of new proteins using this method is contingent upon this condition.

7. Add the stressors (tunicamycin and cycloheximide) to the specifically designated flasks of the resuspended cells; leave the untreated and heat-stressed marked flasks.

*Note: It is crucial to emphasize that the stressors are introduced immediately after the cells are resuspended in AHA-containing standard complete media. The final concentration for tunicamycin treatment is 2.5 μg/mL, while for cycloheximide treatment, it is 50 μg/mL. These stressors are directly diluted from their commercial stock solutions to their final concentrations immediately prior to use.*

8. After that, place all three flasks designated as untreated, tunicamycin-stressed, and cycloheximide-stressed at 30 °C in a shaker incubator at 200 rpm and keep the flask designated as heat-stressed at 37 °C in a shaker incubator at 200 rpm. Incubate for 6 and 24 h, respectively, for short- and long-term stress conditions.

*Note: It is essential to emphasize that all stressor treatments, whether involving heat at 37 °C, tunicamycin, or cycloheximide, are administered continuously for either 6 h or 24 h. Each treatment condition is represented by a single flask. Upon the completion of each time point, normalized cell volumes are extracted from the stressor-treated flasks and the non-stressed flask.*

9. When the incubation is over, normalize the total cell numbers by measuring OD_600_ among the different conditions and then transfer the required volumes of cells to 50 mL centrifuge tubes, spin down at 8,500× *g* for 5 min at room temperature, and harvest for further processing.

**Caution point:** At this juncture, cell normalization is essential to achieve an even distribution of cells for subsequent procedures such as confocal microscopy, flow cytometry, and SDS-PAGE. It is particularly crucial to commence with normalized cell numbers prior to protein extraction; otherwise, there may be variability in protein load across different samples during SDS-PAGE analysis. Cell normalization is typically conducted at a standard target OD_600_ of 1, which is adequate for confocal microscopy and flow cytometry analysis. However, for protein extraction and subsequent SDS-PAGE analysis, it is recommended to begin with at least 20 OD_600_ yeast cells to ensure a sufficient quantity of whole-cell proteins for visualization on gels. If it is not possible to collect 20 OD_600_ yeast cells, normalization should be performed to the highest achievable OD_600_ value.

10. Discard the resultant supernatants, resuspend the pelleted cells in 1 mL of autoclaved water, transfer to microcentrifuge tubes, and centrifuge at 8,500× *g* for 5 min at room temperature.

11. After discarding the supernatant, resuspend the cells in 53% ethanol (v/v) in 1 mL of 1× PBS and keep at 15 °C in a shaker incubator at 200 rpm for 40 min.

**Critical point:** At this stage, the type and concentration of alcohol in PBS, along with the incubation time and temperature in a shaker incubator, are critical factors for achieving a high degree of yeast cell permeabilization. This permeabilization is essential for subsequent evaluation and binding to alkyne fluorophores, which allows for the qualitative and quantitative measurement of new protein synthesis rates.

12. Following this, centrifuge the cells at 8,500× *g* for 5 min at room temperature and discard the resultant supernatants.

13. Resuspend the pelleted cells in 1 mL of the click reaction buffer with the fluorophore that will traverse inside the cells and click it with L-azidohomoalanine incorporated newly synthesized proteins.

**Critical point:** At this stage, maintaining the final concentration of the reaction mixture components is crucial for the successful execution of the Click-it reaction. Failure to do so significantly reduces the probability of obtaining accurate measurements.

**Figure 2.**
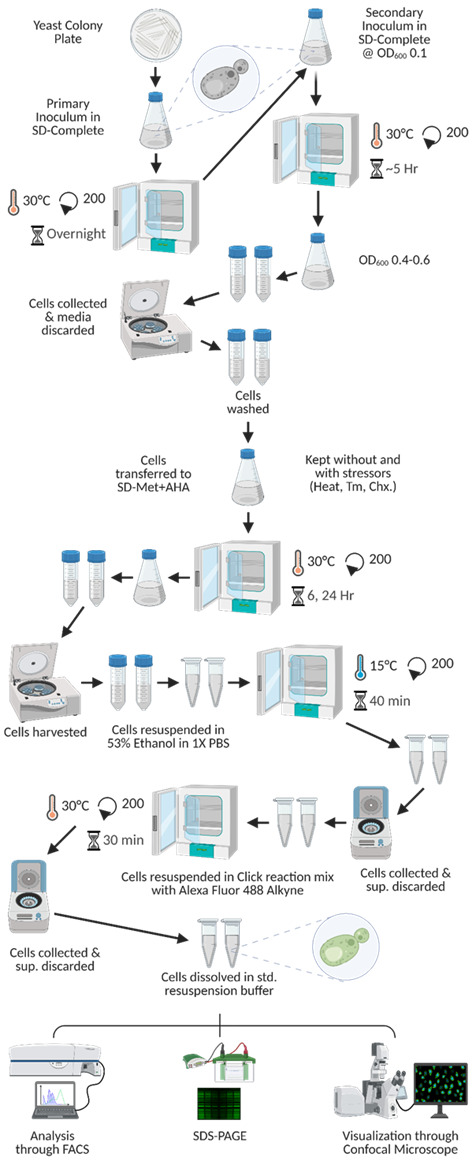
Graphical representation of the procedural workflow of the translation rate determination experiment. In this figure, we detail the chronological processes from start to end for the determination of the rate of new protein synthesis. In the figure, Tm denotes tunicamycin, while Chx refers to cycloheximide. The SD-Met+AHA medium is indicative of SD complete media in which methionine is substituted with L-azidohomoalanine.

14. Keep the resuspended cells at 30 °C in a shaker incubator at 200 rpm for 30 min.

15. Centrifuge the cells at 8,500× *g* for 5 min at room temperature and discard the resultant supernatants.

16. Resuspend the cells in 1 mL of 1× PBS. Take one 10 μL aliquot to visualize the cells through confocal microscopy and another 100 μL aliquot for processing with FACS. Centrifuge the remaining resuspended cells again at 8,500× *g* for 5 min at room temperature, discard the resultant supernatants, and process the cells for protein extraction and SDS-PAGE. Refer to Figure 2 for a visual schematic representation of this workflow.

**B. Confocal microscopy**

1. Before proceeding with confocal microscopy, prepare the 1% agarose pads in 1× PBS on microscopic glass slides in ample numbers and dry them properly. Ensure adequate care to make the pads bubble-free and have the right moisture amount to proceed with microscopy.

**Critical point:** The preparation of agarose pads is crucial for the successful imaging of yeast cells, as these pads serve to immobilize the cells, thereby facilitating proper imaging. However, if the thickness of the agarose pads exceeds the optimal level, imaging becomes challenging, and the resolution and quality of the images deteriorate. Additionally, the moisture content within the agarose pads must be adequate; otherwise, yeast cells may experience osmotic stress, potentially leading to lysis and ultimately resulting in poor-quality microscopic images.

**Figure 3.**
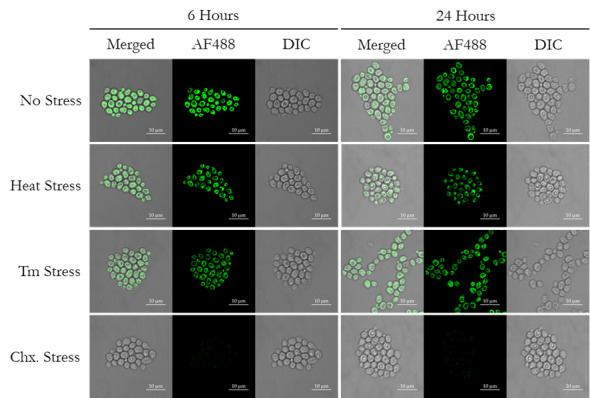
Visualization of the newly synthesized proteins through confocal microscopy. Distribution of newly synthesized proteins in wild-type yeast cells under regular growth conditions (no stress), heat stress, tunicamycin (Tm) treatment, and cycloheximide (Chx., translation blocker, negative control) treatment, at 6 h and 24 h post-treatment.

2. Once the agarose pads are ready, put the separated 10 μL cell aliquots using a pipette onto the pads and place clean microscope glass coverslips on top.

3. Now, as the cells are immobilized in the agarose pads, they are taken for imaging by confocal microscopy.

4. Capture confocal images using the Nikon A1R MP+ Multiphoton Advanced Resolution confocal microscope with the following image capture settings: 100× oil-immersion lens along with the 488 nm solid-state laser, gain 200, offset -25, laser power 5, and TD detector gain 125; the final capture images have another 3-fold zoom, eventually leading the total magnification to 300×. Lastly, all images have a scale bar for comparison of cell sizes.

5. The representative confocal images for the merged channel, along with the single fluorescence and DIC channel, are presented in Figure 3; the left-side and right-side panels show images at 6 and 24 h post-stress, respectively.

**C. Flow cytometry analysis**

**Figure 4.**
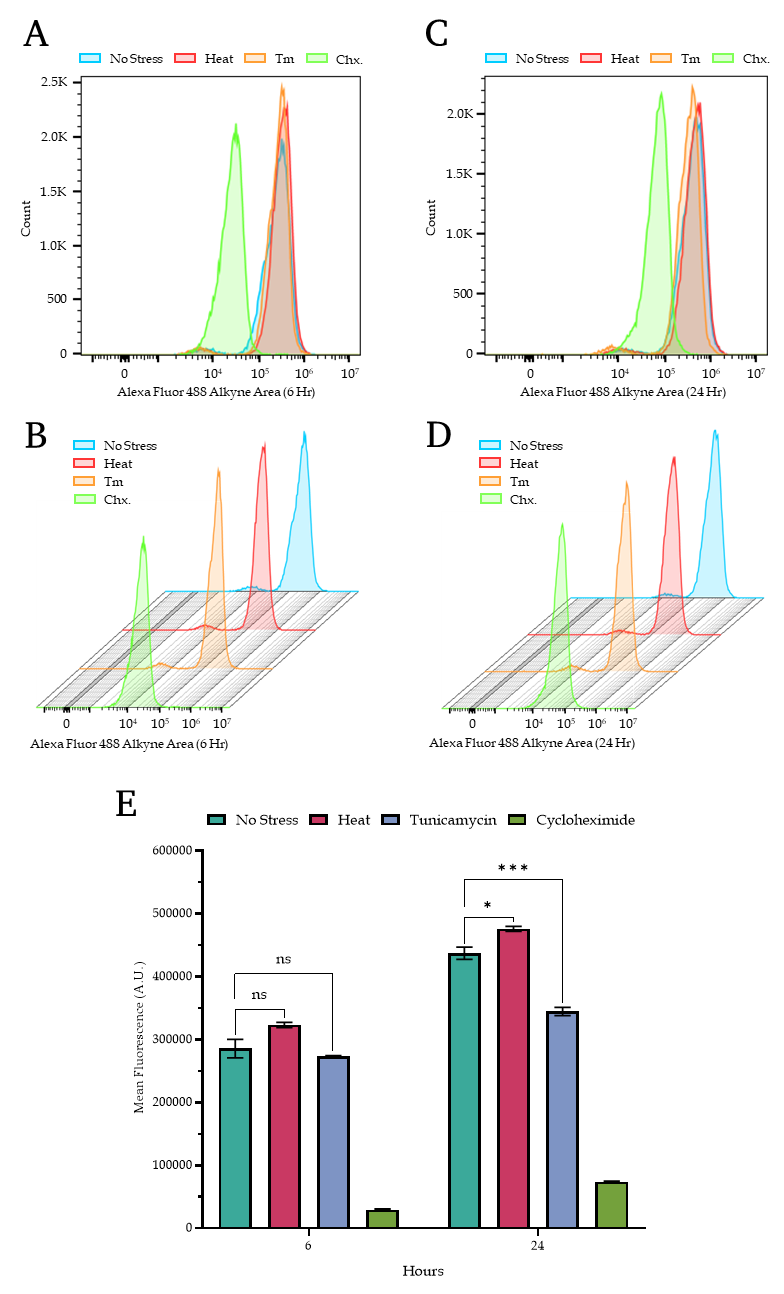
Quantitative measurement of protein translation rate through flow cytometry. (A, B) The overlaid (A) and staggered (B) histogram panels show the fluorescence measurements of wild-type yeast cells corresponding to specific treatment conditions for 6 hours. (C, D) The overlaid (C) and staggered (D) histogram panels show the fluorescence measurements of wild-type yeast cells under similar stress conditions as described in panels A and B for 24 h. (E). Bar plot showing the mean fluorescence intensity of the newly synthesized proteins from wild-type yeast cells under similar stress conditions as described in panel A for 6 and 24 h. The data is the average of three independent replicates, and the error bar represents the standard error of the mean (SEM). The significance is calculated through a T-test, and the signs represent the following: ns – non-significant, * p < 0.05, *** p < 0.001.

**Figure 5.**
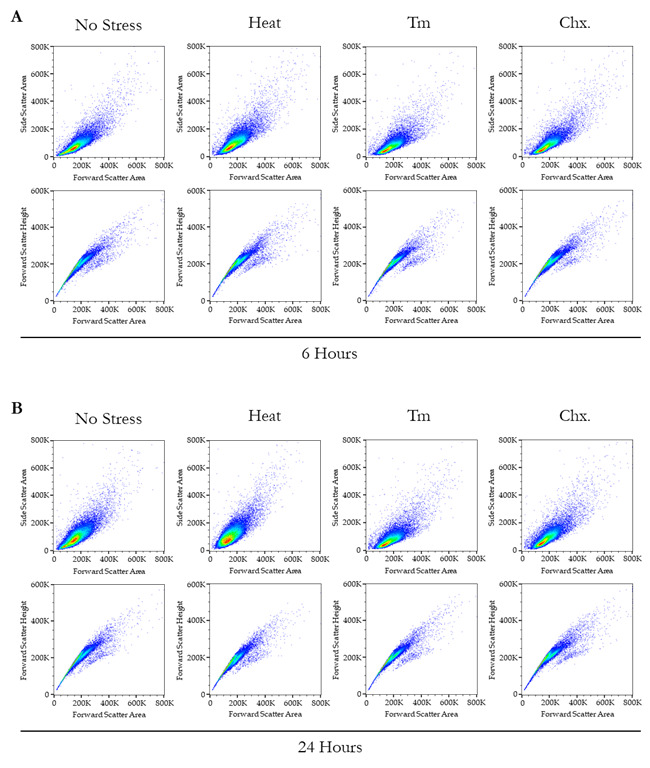
Dot plot parameters for the quantitative assessment of protein translation rates via flow cytometry. (A, B) The scatter or dot plots pertain to the flow cytometry analysis, corresponding to the histograms and bar plot representation presented in Figure 4. In Panel A, the upper scatter plots depict forward scatter–area (FSC-A) on the X-axis and side scatter–area (SSC-A) on the Y-axis under no stress and various stress conditions. Conversely, the lower plots illustrate FSC-A on the X-axis and forward scatter–height (FSC-H) on the Y-axis under no stress and different stress conditions after 6 h. In Panel B, the upper scatter plots represent FSC-A on the X-axis and SSC-A on the Y-axis under no stress and various stress conditions, while the lower plots show FSC-A on the X-axis and FSC-H on the Y-axis under no stress and different stress conditions after 24 h.

1. Load the separated 100 μL cell aliquots in fresh microcentrifuge tubes onto the Beckman Coulter Cytoflex-S cytometer.

2. Set the cytometer acquisition settings for the experiment in the following manner: FSC gain at 45, SSC gain at 40, and Alexa Fluor 488 alkyne gain at 90.

3. For each of the conditions (namely, untreated control, heat, tunicamycin, and cycloheximide), capture a total of 50,000 cells (events).

4. The representative overlayed and staggered histograms from the two different time points are presented in Figure 4, where panel A shows the overlayed histograms from 6 h, panel B shows the staggered histograms from 6 h, panel C shows the overlayed histograms from 24 h, and panel D shows the staggered histograms from 24 h. Panel E shows the bar plot representation of the mean fluorescence intensity from both the 6 and 24 h together. Additionally, Figure 5 provides the complete set of dot plots derived from the flow cytometry data, which correspond to the histograms depicted in Figure 4.

**D. Extraction of proteins and SDS-PAGE**

1. Resuspend the pelleted cells in 600 μL of 0.3 M sodium hydroxide solution and incubate at room temperature in an upright condition for 10 min.

2. Centrifuge the cells at 1,500× *g* for 1 min at room temperature and discard the resulting supernatants.

3. Next, resuspend the pelleted cells in 300 μL of protein storage buffer.

4. Add 300 mg of acid-washed glass beads to each tube.

5. Perform bead beating using the Bead-Ruptor from Omni International while keeping the cycle settings as follows: vortexing intensity at level 3 and time at 3 min.

6. After each 3 min of vortexing, put the respective tubes on ice and incubate for 3 min.

**Critical point:** At this stage, the incubation on ice is essential to mitigate the heat generated within the yeast cell suspension due to vigorous vortexing, thereby aiding in the preservation of the cellular proteins being extracted. If the temperature of the suspension exceeds a certain threshold, the proteins will degrade exponentially with the increase in temperature.

7. Repeat the whole process of vortexing and subsequent ice incubation described in steps D5–6 four times, eventually leading to a total vortexing time of 12 min.

8. Centrifuge the tubes at 16,900× *g* for 5 min at 4 °C to clear out the cellular debris along with the glass beads.

9. Transfer the resultant supernatants to fresh microcentrifuge tubes and centrifuge at 16,900× *g* at 4 °C for 30 min to further clear out insoluble protein aggregates and other debris.

10. Transfer the resultant supernatants to fresh microcentrifuge tubes; this would be the whole cell protein extracts. Beyond this point, always keep the protein extracts on ice or at 4 °C for immediate use; alternatively, store at -20 °C for later use.

**Pause point:** At this juncture, the isolated proteins may be preserved for subsequent utilization in the SDS-PAGE analysis.

**Figure 6.**
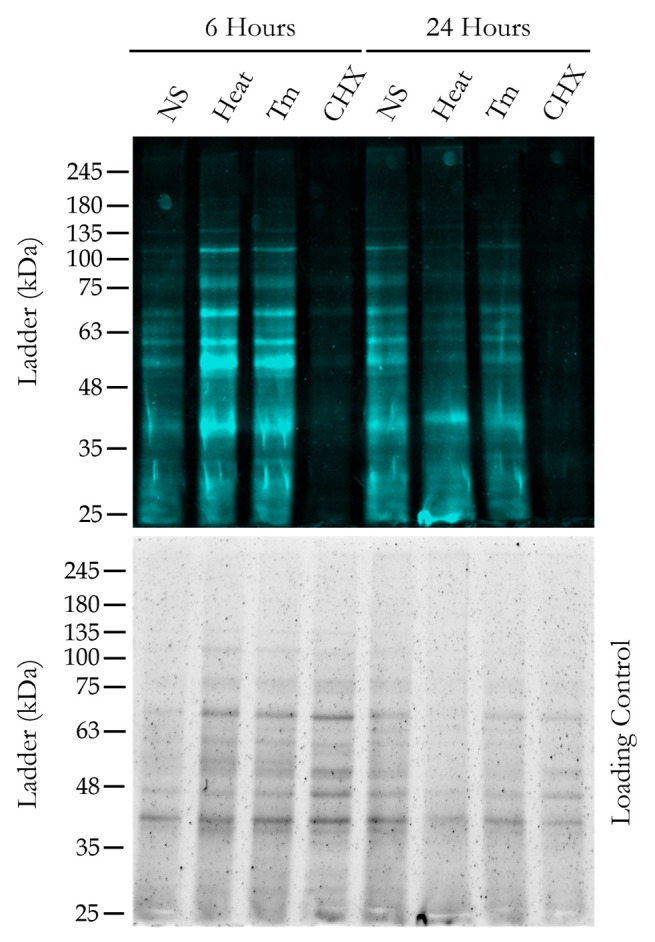
Visualization of newly synthesized proteins after gel electrophoresis. The top panel shows the newly synthesized proteins extracted from wild-type yeast cells, detected under the fluorescence scanner (488 nm excitation/515 nm emission) of the gel documentation system (iBright Imaging system, Thermo Scientific) after running an SDS-PAGE under specific treatment conditions for 6 and 24 h. To exhibit the total protein load in each lane, a whole gel loading control image was taken under the UV scanner of the gel documentation system after the SDS-PAGE (bottom panel). The protein ladder used for SDS-PAGE is from GeneDirex (catalog number: PM007).

11. Measure the protein concentrations for each tube representing different stress conditions through the BCA protein estimation method (quantitation not shown).

12. Depending on the respective protein concentrations of each treatment condition, mix the required volumes of protein extracts with 5× SDS loading dye such that the final concentration of loading dye would be 1× in all samples.

13. Boil the SDS loading dye mixed samples at 95 °C for 15 min with intermittent vortexing.

14. Centrifuge the samples at 20,000× *g* for 2 min at room temperature to clear out the debris.

15. At this point, prepare a 10% polyacrylamide gel using standard molecular biology laboratory protocols. Add a stain-free imaging chemical for proteins to better visualize the total protein load in the whole gel after the electrophoresis run under UV light.

16. When the gel is ready, load 10 μL from the top layer of each of the boiled protein samples onto a single lane of the gel.

17. Perform the electrophoresis run at a constant 100 V until the dye front moves out of the gel.

18. Expose the gel under UV light for 5 min to induce the chemical to form an adduct with the proteins in the gel.

19. Visualize the gel through the iBright gel documentation system from Invitrogen with 488 nm fluorescence excitation and a subsequent detection system. The resultant image shows the newly synthesized proteins in green fluorescence bands. A representative image is presented in Figure 6 (top panel).

20. Visualize the gel through the Bio-Rad EZ gel documentation system with UV excitation and a subsequent detection system. The resultant image shows the total normalized protein load in each lane of the gel. The representative image of the same is presented in Figure 6 (bottom panel).

## Data analysis

**Confocal microscopy:** Confocal microscopy images were obtained utilizing Nikon NIS-Elements AR 5.20.02 software and preserved in the native .nd2 format. To prepare images for figure presentation, denoising and deconvolution procedures were applied within the software environment, followed by exportation as .tiff files.

**Flow cytometry analysis:** Flow cytometry data were collected using Beckman Coulter CytExpert 2.5.0.77 software and initially saved in the .xit format. For subsequent analysis and figure generation, data were converted to .fcs format, with measurement parameters exported as .csv files. The FlowJo V10.8 platform was employed to create overlaid and staggered figure panels from .fcs files. Microsoft Excel was then used to format raw measurements and calculate mean fluorescence values for plotting purposes. Additionally, multiple T-test analyses were performed within Excel. The final bar plot was generated using GraphPad Prism V 9.5.0, incorporating the reformatted mean fluorescence intensity data and T-test results from Excel.

**Gel imaging:** Gel visualization was conducted using two methods. First, fluorescence imaging was performed with the iBright Gel-Doc system from Invitrogen. Subsequently, UV imaging was carried out using the Bio-Rad EZ Gel-Doc. Both instruments produced high-resolution .tiff images, which were incorporated into the appropriate figures.

**Results**

Our primary objective with this study was to successfully and efficiently measure the rate of new protein translation in our budding yeast *Saccharomyces cerevisiae* BY4741 strain, which usually serves as a wild-type strain. We have already discussed in great detail how to prepare all the necessary media, reagents, and buffers and how to process the yeast cells to measure the translation rate. In this section, we are discussing the results obtained through our formulated protocol.

**1. Visualization of newly synthesized proteins.** We first wanted to validate that the newly synthesized proteins in our yeast strain have incorporated the methionine analog L-azidohomoalanine, which can be detected through the Click chemistry–based reaction using any alkyne-modified fluorophore. In our experiment, we used Alexa Fluor 488 alkyne. To visualize the cells, we pelleted the cells, resuspended in 1× PBS, placed them on agarose pads, and imaged them under a confocal microscope. As can be seen in Figure 3, we imaged the cells at 6 and 24 h to visualize the newly synthesized proteins within the cell and their distribution. We kept one control condition where the cells are growing regularly, one condition with heat stress at 37 °C, one condition with tunicamycin (Tm 2.5μg/mL) stress, which activates the endoplasmic reticulum-unfolded protein response (ER-UPR) pathway, and a last condition with cycloheximide, which acts as a protein translation blocker, serving as a negative control. It can be clearly seen in Figure 3 that in the cycloheximide-treated cells, no new protein translation was evident, as expected; thus, there is no L-azidohomoalanine incorporation, hence no fluorescence was detected post-Chx treatment. In the case of regular growth conditions, we see significant amounts of L-azidohomoalanine incorporation, as visualized by Alexa Fluor 488 alkyne fluorescence intensity. But if we compare the heat and Tm stress conditions to untreated regular growth conditions, we can see that there is variation in fluorescence intensity, signifying a variable protein synthesis rate corresponding to the stress treatment. As the measurement of protein synthesis rate from fluorescence images could be tricky, laborious, and error-prone, we proceeded to perform flow cytometry measurements to have a better quantitative assessment of the AHA-incorporation during protein translation.

**2. Quantitative measurements of new protein translation rate.** After visualization through confocal microscopy, we measured the fluorescence intensity quantitatively with the help of flow cytometry. The detailed parameters for flow cytometric evaluations were described in the methodology section, and we kept the same treatment conditions along with the same controls at the same time points as described in the confocal microscopy visualization (6 and 24 h; no-stress, heat stress, Tm-stress, and cycloheximide treatment conditions). The measurements can be seen in Figure 4, where the top panels show overlaid histograms, and the middle panel shows staggered histograms of newly synthesized protein measurement values. In Figure 4 (bottom panel), the bar chart shows the quantitative measurements across different treatments along with their significant differences among the two different time points. From the bar chart, it can be concluded that at the end of 6 and 24 h, the new protein translation rate significantly increases in the case of heat stress conditions, when compared to regular growth conditions. In the condition treated with tunicamycin, the translation rate exhibits a marginal decrease after 6 h, although this change is statistically insignificant. However, after 24 h of tunicamycin treatment, there is a marked reduction in translation in the BY4741 strain compared to non-stressed, regular growth conditions. After this, we proceeded to extract the whole cell protein extract from the yeast strain and performed SDS-PAGE.

**3. Protein extraction, SDS-PAGE, and fluorescence visualization.** After the flow cytometry analysis, we extracted the whole cell proteins and performed SDS-PAGE for the same strain under the same growth and treatment conditions. After the gel run was complete, the gel was visualized under a gel documentation system with a fluorescence scanner. As can be seen in Figure 6, at the end of 6 and 24 h, the fluorescently labeled newly synthesized proteins can be clearly seen in no-stress, heat, and Tm stress conditions, whereas in cycloheximide stress, as there is no new protein synthesis, there is no significant fluorescence detected. We also kept the whole gel protein loading control for reference.

**Discussion**

Our research primarily aimed to develop and formalize a systematic approach for quantifying the rate of protein synthesis in *Saccharomyces cerevisiae*, commonly known as budding yeast. The application of flow cytometry, confocal microscopy, and SDS-PAGE methodologies demonstrated efficacy in the detection and quantification of newly synthesized proteins within yeast cellular systems, as evidenced by our investigative results. While protocols for assessing protein synthesis rates in mammalian cells have been extensively documented in prior research, a notable absence of clearly defined and uniform methodologies persists for yeast cells. Elucidating the rate of protein synthesis in *Saccharomyces cerevisiae* is of paramount importance for diverse applications in biotechnology and biomedical investigations. The experimental protocol employed in this investigation demonstrated enhanced precision and selectivity in identifying newly formed proteins within yeast organisms, surpassing the capabilities of current protocols designed for mammalian cellular systems. Notwithstanding certain reports that suggest limitations in the utilization of flow cytometry for yeast analysis, our experimental outcomes indicate that this technique represents a feasible approach for such investigations. Our findings are substantiated by a comprehensive array of analytical techniques, including flow cytometry analysis, confocal microscopy imaging, and fluorescence gel visualization of SDS-PAGE outcomes. The present investigation yields a noteworthy advancement in the field of yeast biology by introducing a dependable technique for assessing protein synthesis rates. The standardized methodology demonstrates applicability across diverse research contexts, including pharmaceutical development and metabolic engineering, enabling the investigation of protein synthesis processes in yeast systems. Although our methodology demonstrates robustness, it is imperative for researchers to meticulously calibrate their equipment to mitigate potential inaccuracies in measurements. This research represents the pioneering effort to elucidate a well-defined and standardized protocol explicitly tailored for assessing the rate of cellular translation in the model organism *Saccharomyces cerevisiae*, more commonly referred to as budding yeast. The implementation of our study protocol is constrained by its reliance on specialized instrumentation and technical proficiency, which may pose accessibility challenges for certain research groups.

## Validation of protocol

From the initial stage, we maintained three independent biological replicates of our yeast strain for both primary and secondary inoculation, which were consistently utilized across various experimental setups, including confocal microscopy, flow cytometry analysis, and protein extraction followed by SDS-PAGE analysis. In the confocal microscopy procedure, we captured five distinct fields for each replicate, resulting in a total of 15 microscopic image fields per treatment condition. For flow cytometry analysis, three individual replicates were retained for each treatment condition, with 50,000 cells (events) recorded per vial, culminating in a total of 1,50,000 cells for each treatment condition. In the whole-cell protein extraction and SDS-PAGE analysis, three individual replicates were maintained for each treatment condition, with only one replicate presented in Figure 5 for illustrative purposes. Statistical significance was assessed using a T-test for pairwise comparisons between treatment conditions and untreated controls. The significance indicators and their corresponding values are detailed in the figure legends, where the statistical tests are depicted within the plots. In this experimental protocol aimed at determining the translation rate of actively growing yeast cells, unstressed cells served as a positive control, where protein synthesis occurred at a natural rate, while cycloheximide was employed as a negative control to completely inhibit translation. The data presented in the figures confirm that the positive control exhibits translation, whereas the negative control demonstrates a cessation of translation.

This protocol (or parts of it) has been used and validated in the following research article:

• Jha et al. [6]. Sse1, Hsp110 chaperone of yeast, controls the cellular fate during endoplasmic reticulum stress. G3 – Genes, Genomes, Genetics (Figure 3b).

## General notes and troubleshooting

**General notes**

1. The success of this protocol lies in the fact that the media wherein the yeast cells are growing does not contain any methionine; instead, it contains L-azidohomoalanine. So, when new proteins are synthesized, they will contain L-azidohomoalanine instead of methionine, and those will be detected using alkyne-modified fluorophore through click reaction. So, make sure there is no presence of methionine in your media.

2. Make sure to keep L-azidohomoalanine in the same proportion as for methionine in the standard media for yeast.

3. Make sure to grow the cells until their log phase in standard media and then transfer to L-azidohomoalanine-containing media; start any stressor treatment accordingly to the respective experimental setup.

4. After the desired treatment period, wash the cells for any residual media and then permeabilize the cells properly as described in this protocol. Otherwise, the resulting data would be erroneous and hard to reproduce.

5. For capturing microscopic images, the cells must be immobilized in agarose pads. Ensure extreme care while preparing agarose pads and make their thickness even and as thin as possible while maintaining enough moisture to proceed to microscopy.

6. For flow cytometry analysis, limit the cell density to a moderate level. Excessively dense cell suspensions might lead to erroneous results.

7. For protein gel visualization after SDS-PAGE, use the fluorescence-activated gel doc system; otherwise, newly synthesized proteins will not be distinctly visualized.

**Troubleshooting**

Problem 1: The fluorescence intensity is very low in confocal microscopy and flow cytometry measurements.

Possible cause: The cells might not be permeabilized properly.

Solution: Ensure great care while permeabilizing the cells and strictly follow the buffer composition, treatment temperature, and shaking conditions.

Problem 2: The determined translation rate is similar in the positive and negative controls, or the negative control shows L-azidohomoalanine incorporation.

Possible cause: The cycloheximide treatment did not work properly.

Solution: Make sure the cycloheximide is freshly prepared and in its active state, and ensure the cycloheximide treatment is given properly to the cells.

## References

[1] Ingolia N. T., Hussmann J. A. and Weissman J. S. (2019). Ribosome Profiling: Global Views of Translation. Cold Spring Harb Perspect Biol. 11(5): a032698. https://doi.org/10.1101/CSHPERSPECT.A032698 PMC6496350 30037969

[2] Hershey J. W., Sonenberg N. and Mathews M. B. (2012). Principles of Translational Control: An Overview. Cold Spring Harb Perspect Biol. 4(12): a011528. https://doi.org/10.1101/CSHPERSPECT.A011528 PMC3504442 23209153

[3] Dykeman E. C. (2023). Modelling ribosome kinetics and translational control on dynamic mRNA. PLoS Comput Biol. 19(1): e1010870. 10.1371/JOURNAL.PCBI .1010870 PMC9894550 36689464

[4] Jia X., He X., Huang C., Li J., Dong Z. and Liu K. (2024). Protein translation: biological processes and therapeutic strategies for human diseases. Signal Transduct Target Ther. 9(1): 44 10.1038/S41392-024-01749-9 38388452 PMC10884018

[5] Birhanu A. G. (2023). Mass spectrometry-based proteomics as an emerging tool in clinical laboratories. Clin Proteomics. 20(1): 1 20 20. 10.1186/S12014-023-09424-X/FIGURES/2 37633929 PMC10464495

[6] Jha M. P., Kumar V., Ghosh A. and Mapa K. (2024). Sse1, Hsp110 chaperone of yeast, controls the cellular fate during endoplasmic reticulum stress. G3(Bethesda). 14(6). 10.1093/G3JOURNAL/JKAE075 PMC11152076 38577891

[7] Trawczyńska I. and Wójcik M. (2015). Optimization of permeabilization process of yeast cells for catalase activity using response surface methodology. Biotechnol Biotechnol Equip. 29(1): 72 10.1080/13102818.2014 .934986 26019618 PMC4434045

